# Lateral Window Design for Maxillary Sinus Graft Based on the Implant Position

**DOI:** 10.3390/ijerph17176335

**Published:** 2020-08-31

**Authors:** Kyeong-Jun Cheon, Byoung-Eun Yang, Seoung-Won Cho, Sung-Min Chung, Soo-Hwan Byun

**Affiliations:** 1 Department of Oral & Maxillofacial Surgery, Dentistry, Sacred Heart Hospital, Hallym University College of Medicine, Anyang 14068, Korea; shgusbabo@naver.com (K.-J.C.); face@hallym.or.kr (B.-E.Y.); kotneicho@gmail.com (S.-W.C.); 2 Research Center of Clinical Dentistry, Graduate School of Clinical Dentistry, Hallym University, Chuncheon 24252, Korea; 3 R&D Center, Genoss, Suwon 16229, Korea; soodentist@naver.com

**Keywords:** maxillary sinus augmentation, lateral window design, position, implant

## Abstract

The purpose of this study was to devise a classification and lateral window design method based on implants and to evaluate whether these classifications and methods are applicable to clinical practice. When applying the maxillary sinus elevation technique using the lateral window, possible situations were classified into four: (A) two or more sites for implants are required for maxillary sinus augmentation, (B) a single implant is required when there are no adjacent teeth, (C) a single implant is required when one adjacent tooth is present at the mesial or distal area, and (D) a single implant is required when both mesial and distal adjacent teeth are present. In order to verify whether this classification can be used in all situations, 76 patients who underwent maxillary sinus elevation with a lateral window were selected and investigated. Of them, 47 (62%) were included in Group A, 9 (12%) in Group B, 8 (11%) in Group C, and 12 (15%) in Group D. Lateral window designing in the lateral approach of sinus augmentation can be classified into four clinical situations. There were no unclassified cases. This classification and window positioning method can be applied to most cases.

## 1. Introduction

The maxillary posterior edentulous region is an area where implant placement is difficult due to its anatomical characteristics, and the survival rate of the implant is low due to the bone quality and quantity [1,2,3]. One reason for the low survival is that it is adjacent to the surgical site, making it difficult to secure the residual bone mass. In cases of edentulous patients, maxillary sinus pneumatization is accelerated and the residual bone can decrease to less than 1 mm. A second reason is that the cortical bone is thinner than the mandibular bone and the density of the spongy bone is low, which makes the bone quality unfavorable for implant [4,5,6,7]. The alternative way is to perform a maxillary sinus bone graft using maxillary sinus augmentation. There are two main approaches for maxillary sinus floor elevation: the crestal and lateral approach. The crestal approach was first performed by Tatum in 1981 [8]. The Caldwell-Luc approach is another way to perform maxillary sinus bone graft using maxillary sinus augmentation, as described in 1980 by Boyne and James [9]. In that procedure, a window is opened on the side wall bone of the maxillary sinus, lifted from the sinus membrane, and grafted by crushing the bone collected from the long bone between the mucosa and the bone. Since then, a number of papers on maxillary sinus surgery have been published, and many new instruments and technologies have been developed. The goals for the sinus augmentation procedure is usually to prepare the site for implant placement and restore deficiency. Most papers report success rates according to the implant length, type, or type of bone graft material placed after maxillary sinus augmentation [2,10,11]. The studies on the maxillary sinus grafting technique mostly focus on the differences between and criteria for the lateral approach and the crestal approach [12].

In terms of instrument development, the piezoelectric technique was introduced in 2001, as a window forming method [13], and the various lateral approach technique was introduced by many implant companies [14]. A recently introduced technique for osseodensification of dental implants involves the use of special drills running in a counterclockwise direction at the implant site [15]. In the case of the advancement of technology, there is a main focus on reducing the size of the window using minimally invasive methods and the development of bone graft materials. However, few studies have been conducted on the positioning of the lateral window, which is the most important factor in maxillary sinus augmentation [16,17]. Eliaz reported that the lower border of the osteotomy should be approximately 3 mm above the sinus floor, the osteotomy should be oval or rectangular, and its corners and sharp edges should be avoided to minimize the risk of tearing the sinus membrane [17]. However, there are several drawbacks in this method. First of all, maxillary sinus surgery is a technique for implant placement, but there is no consideration for implant placement. Because there is no visible landmark as a reference, the operator often intuitively decides on the window position during the procedure rather than planning in advance. In order to overcome this limitation, surgery using a surgical guide has recently been introduced [18,19]. This is one of the main reasons for the difficulty in lateral window procedures because high sensitivity in the operator’s skill is needed.

Gustavo et al. presented that the smaller the lateral window dimension, the more the maturation and consolidation of the mixture of cortical and cancellous allograft of the bone [20]. Most previous studies have commented only on the basic principles such as the anatomical criteria of the maxillary sinus or prevention of tearing or bleeding of the membrane. However, it is not easy to apply these methods ideally in clinical practice. Maxillary sinus augmentation is a technique for implant placement; however, existing methods, which do not consider the position of implants, have some limitations. Setting the window position without any specific classification leads to the use of ambiguous words such as “approximately,” or “as small as possible.” In previous studies, the window size was more of a focus than the window position and studies focused on the classification of sinus membrane perforation during augmentation procedures [21]. In order to solve these problems, this study recommends standards by setting a classification and detailed rules for lateral window positioning. This classification allows a quick and precise lateral window positioning and also permits those who are unfamiliar with the procedure to be trained. In addition, it will be helpful in setting the position of the window as seen in the recent surgical guide. The purpose of this study is to propose a method to determine lateral window position and verify its effectiveness in various clinical cases.

## 2. Material and Methods

The ethics committee of the Hallym University (IRB No. 2020-05-010) approved the use of this data. Since this was a retrospective study, written informed consent was waived. One clinician reviewed the medical records of all patients who visited the Hallym University Medical Center and Well Dental Clinic from January 2015 to December 2019, underwent maxillary sinus augmentation via the lateral approach, and completed the second implant surgery and crown prosthesis treatment. We included all patients who underwent implantation with a simultaneous maxillary sinus augmentation using the lateral approach.

**Figure 1:**
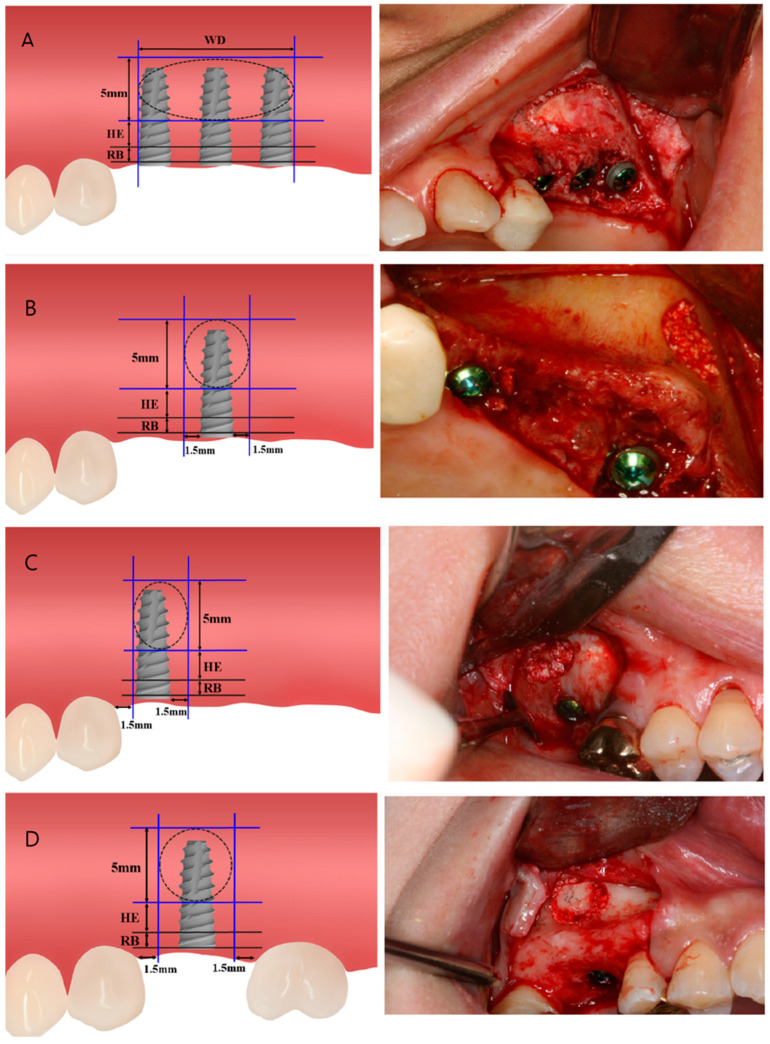
Diagram of the classification and related clinical photographs. (**A**) Group A. (**B**) Group B. (**C**) Group C. (**D**) Group D. RB (residual bone height): distance from alveolar ridge to inferior border. HE (vertical height for inferior border of lateral window): 3 mm when the residual alveolar bone height was 3 mm or more, and 5 mm when the residual bone height was less than 3 mm. WD (width for horizontal border of lateral window): medial border to the distal margin of the implants.

The patients were classified into four clinical situations. Group A included cases with more than two implants. Group B included cases with a single implant without any adjacent teeth. Group C included cases with a single implant and a single adjacent tooth at the mesial or distal area, and Group D included cases with one implant with adjacent teeth at the mesial and distal areas (Table 1, Figure 1).

A clinician reviewed the treatment plans, including the location and the number of implants, using panoramic radiographies and cone beam CT before the operation. The height of the residual bone was evaluated; if it was 5 mm or less, maxillary sinus augmentation by the lateral approach was performed. In addition, the classification method was applied according to the existence of adjacent teeth and the number of implants placed. The examiner who evaluated all the patients in this study was a skilled surgeon with extensive experience in implantology.

After the cases were classified according to the method described above, the position of the window was set according to the following detailed rules: The lateral window could be made into a rectangle or a circle in a rectangular frame by drawing four lines on the mesial, distal, superior, and inferior sides. In Group A, the mesial boundary of the lateral window was set as the mesial border of the most mesially located implant fixture, and the distal boundary was decided as the distal border of the most distally located implant fixture. In the cases of Group B, C, and D, the mesio-distal boundaries were 1.5 mm away from the adjacent teeth or implants, if there were adjacent teeth and implants. The lower boundary of the window was placed 5 mm above the inferior border of the maxillary sinus when the residual bone length was less than 3 mm, and 3 mm above the inferior border of the maxillary sinus when the residual bone mass was 3 mm or more. The upper boundary of the window was 5 mm above the decided lower boundary (Figure 1).

## 3. Results

A total of 135 implants were implanted. Thirty-eight cases (50%) underwent window opening procedures using a low-speed handpiece, 14 cases (18%) used a high-speed handpiece, and 24 cases (32%) used a New Sinus kit. There were 52 cases (68%) in which the bony windows were repositioned, and 24 cases (32%) in which the repositioning procedure was not performed. There were 47 patients (62%) in Group A, 9 (12%) in Group B, 8 (11%) in Group C, and 12 (16%) in Group D (Table 1). There were 42 (55%) male patients and 34 (45%) females. All identified patients could be assigned to the groups and there were no cases not covered by the classification.

## 4. Discussion

Simultaneous implant placement with maxillary sinus augmentation using the lateral approach could be used to achieve a proper initial stability [22,23]. The maxillary sinus augmentation technique is largely divided into a lateral approach method to form a window on the side wall bone of the maxillary sinus and lift the sinus membrane, and a method to be performed at the extraction or alveolar crestal site. Crestal approach technique starts with preparing the implant site. A green-stick fracture of the sinus floor was performed by hand or electric tapping of the socket in a vertical direction until a fracture of the sinus floor was obtained [24]. The stability and reliability of the crestal approach has been indicated by many studies [25,26]. Roberto C. et al. performed a retrospective study and confirmed the reliability of the transcrestal sinus floor elevation procedure and the maintenance of the bone levels without grafting procedures over time [27]. Compared to the crestal approach, the lateral approach is difficult and complicated. Recently, a published study used hydraulic pressure in the lateral approach to lift the maxillary sinus, which was used only in the crestal approach [28]. In addition, osseodensification techniques for crestal sinus lifting was published recently, and its stability was verified in a 5-year follow-up study [15].

Tiziano et al. reported that the ideal location for the window is 3 mm superior to the sinus floor and 3 mm distal to the sloping anterior wall, which allows controlled membrane augmentation while keeping the elevating instruments on the bone surface at all times [4]. George et al. revealed that the 3D surgery guide for maxillary sinus grafting was made 3 mm upward along the outline of the maxillary sinus and considered it as the basis for the lower baseline of the new positioning criteria in this study [16]. Pariente et al. reported that the ovate shape of the SCC4 instrument (EBI Sinus Kit, EBI North America^®^, NJ, USA) was used to draw the windows in the lateral wall of the sinus. It has an elliptic shape 5 mm high and 8 mm long [29]. Based on these studies, the upper boundary line was decided 5 mm above the lower boundary line. In previous studies, the criteria for positioning the window were based on the outline of the maxillary sinus without any other specific basis. This method is difficult to apply clinically because there is no measurable landmark visible on the bone wall of the maxillary sinus, even after periosteal elevation.

**Figure 2:**
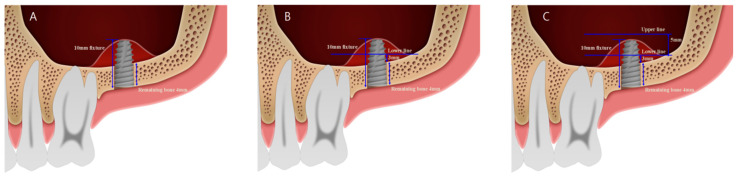
Diagram of volumetric analysis. (**A**) 10-mm long fixture; the remaining bone is 4 mm; the 6-mm part of the fixture would be exposed. (**B**) The lower line could be drawn 3 mm above the inferior border of the maxillary sinus. (**C**) The superior line could be drawn 5 mm above the first inferior boundary of the lateral window.

When considering the criteria to position the lateral window, the number of implants and adjacent teeth/implant should be of primary concern, rather than the outline of the maxillary sinus, for ideal maxillary sinus augmentation and implant placement. This study demonstrated that it is important to set a boundary at the mesial, distal, superior, and inferior sides. For example, boundaries at the mesial and distal sides could be determined through adjacent teeth/implants. The superior boundary was set 5 mm upward from the inferior reference line, considering the mean absorption of bone graft material and sufficiency of space. According to Klijin et al., the amount of bone graft absorbed after maxillary sinus augmentation is averagely 25% for 12 months, and an average height of 2 mm was lost [30]. For example, when a 10-mm long fixture is placed in a case where the remaining bone is 4 mm, the 6-mm part of the fixture would be exposed in the maxillary sinus (Figure 2A). According to the criterion, the lower line of the lateral window could be drawn 3 mm above the inferior border of the maxillary sinus (Figure 2B) and the superior boundary of the lateral window could be drawn 5 mm above the first inferior boundary of the lateral window (Figure 2C). The 6-mm exposed part of the fixture would be covered with 8 mm of the grafted bone in the maxillary sinus. Even if 25% (2 mm) of bone absorption occurred, the exposed part of the fixture would be surrounded with the remaining grafted bone. When vertical lines were decided on both sides (mesial/distal), the distance of vertical line to adjacent teeth was set at 1.5 mm to prevent damage to the adjacent teeth during the formation of the lateral window. Misch et al. suggested that it is better to reduce the distance to more than 1.5 mm from the adjacent teeth and 3 mm or more between the implants during implant placement [31]. Berglundh et al. recommended to leave 1.5 mm between the tooth and implant to maintain the bone adjacent to the teeth and to obtain good esthetic results [32]. A lower horizontal line was set according to the residual bone height, which is an important factor for the implant survival rate. If the residual bone height is short, bone fractures may occur during window opening procedure in maxillary sinus augmentation or implant drilling. Ofer et al. reported three out of 88 clinical cases of maxillary sinus augmentation with bone fracture during implantation with simultaneous sinus augmentation in 1-3 mm residual bone [22]. Wang et al. proposed a simple ABC classification system to guide clinicians to properly treat implants. According to the ABC classification, Class C which is difficult to treat indicates less than 5-mm bone height below the sinus floor [33]. Accordingly, when the residual bone height was 3 mm or less, the lower line should be placed at 5 mm above the lower border of the maxillary sinus.

A limitation in our procedures is that lesions or masses in the maxillary sinus, a thick/thin sinus wall, blood vessels, and a maxillary sinus septum at the site of the lateral window may not enable the fully application of our criteria. Mardinger et al. stated that the prevalence of maxillary sinus lesions was 1.4% to 9.6%, but most of these lesions were not a contraindication to maxillary sinus surgery [34]. The arterial vascularization of the maxillary sinus is divided into three main arteries: the posterior lateral nasal artery, infraorbital artery, and posterior superior alveolar artery. The vascularization of the anterolateral wall of the sinus is characterized by the presence of the alveolar antral artery, an intraosseous anastomosis between the dental branch of the posterior superior alveolar artery and the infraorbital artery [4]. Rossano et al. reported that the intraosseous anastomosis courses halfway up the lateral sinus wall and is always present in the cortical bone of the lateral wall of the maxillary sinus [35]. Tiziano et al. stated that the blood vessels causing bleeding in the maxillary sinus area need to be considered if their diameter is greater than 2 mm, which occurs in about 2% of cases [4]. The presence of blood vessels could influence the decision of the lateral window position [36]. Some practitioners use piezosurgery to create a window and avoid blood vessel damage in the presence of blood vessels; however, many practitioners establish an ideal lateral window position and create a lateral window in the area even if there are blood vessels. Once hemostasis is achieved, you can proceed to the next step. Therefore, the blood vessels do not really affect the classification in this study. The new Sinus kit can be utilized in thick walls and high-speed diamond burs can be used in thin walls. The thickness did not affect the window positioning, only the window opening method. The maxillary sinus septum usually originates from the sinus floor and may stretch for a variable height on the lateral wall. The septa consist of a bone cortex, usually oriented in the vestibular-palatal direction, and divides the distal part of the sinus into multiple compartments known as posterior recesses [4]. If sinus septa are present, it could be dealt with by preparing separate osteotomies on either side of the septum. Krennmair et al. reported a prevalence of up to 58% for maxillary sinus septa. The study recommended that clinicians look for the existence of the septum before planning for surgery [37].

Except for very few unusual cases, the classification in this study can be applied in positioning the lateral window during sinus augmentation. This classification will improve the reproducibility and accuracy of the procedure and facilitate communication with an inexperienced operator, in a teaching setting. In addition, with the recent development of digital dentistry using 3D imaging, it is possible that this classification is a reference point in establishing a guide for maxillary sinus surgery [19]. In designing the lateral window using this classification, it is expected that a surgical guide will be easier to manufacture and its accuracy will be improved. This new criterion does not require any additional step but rather simplifies and improves the accuracy of the procedure. Furthermore, the classification in this study will be helpful to establish the ideal implant prosthesis. In the future, additional research and data collections using exceptional cases are required.

## 5. Conclusions

Four classifications were devised to determine the lateral window positioning during maxillary sinus elevation. This classification was applicable to all clinical cases in this study and can ensure the prediction of an accurate window design. Lateral window opening could be performed quickly, easily, and safely during the implant surgery in sinus augmentation by using this classification.

## Figures and Tables

**Table 1:** Classification of maxillary sinus augmentation.

Classification	Number of Implants	Adjacent Tooth	Number of Cases (%)
Group A	>1	Not related	47 (62%)
Group B	1	None	9 (12%)
Group C	1	Either one	8 (11%)
Group D	1	Both	12 (15%)
